# Paroxetine-induced black hairy tongue and necrotizing sialometaplasia: a case report

**DOI:** 10.1186/s13256-025-05061-0

**Published:** 2025-11-28

**Authors:** Zhuang Ying Lee, Manjit Dhillon

**Affiliations:** Aberdeen Royal Infirmary, Foresterhill, Aberdeen, AB25 2ZN UK

**Keywords:** Paroxetine, Selective serotonin reuptake inhibitor, Black hairy tongue, Necrotizing sialometaplasia

## Abstract

**Background:**

Black hairy tongue, also known as lingua villosa nigra, is a reversible benign disease on the dorsum of the tongue characterized by elongated and hypertrophied filiform lingual papillae. Necrotizing sialometaplasia; on the other hand, is a benign reactive inflammatory disorder involving a minor salivary gland. The pathophysiology driving black hairy tongue and necrotizing sialometaplasia remains unclear; however, common predisposing factors for both conditions include smoking, alcohol, and drugs, and in this case, paroxetine, a selective serotonin reuptake inhibitor.

**Case presentation:**

A 27-year-old Caucasian (British) lady presented with a 4-month history of recurrent ulcerations on her soft palate a few months after she was commenced on paroxetine for her mental health. The lesions were managed conservatively. Following a quiescent period of 19 months, she presented with a very inflamed and ulcerated palate, with heavy deposits of debris on a brown hairy tongue. Her mental health did not improve with paroxetine; hence, paroxetine was stopped and she was started on a different class of antidepressants. Both of her oral lesions markedly improved following the withdrawal of paroxetine.

**Conclusion:**

This case demonstrates the need to consider black hairy tongue and necrotizing sialometaplasia as differential diagnoses in patients with relevant risk factors presenting with oral lesions. Black hairy tongue and necrotizing sialometaplasia can both be managed conservatively by removal of the causative factor and maintaining good oral hygiene; nevertheless, timely and accurate diagnosis is important owing to the fact that necrotizing sialometaplasia may closely resemble more sinister pathology. Clinicians may also consider including black hairy tongue and necrotizing sialometaplasia as rare side effects of paroxetine when offering medication counseling.

**Supplementary Information:**

The online version contains supplementary material available at 10.1186/s13256-025-05061-0.

## Introduction

Selective serotonin reuptake inhibitors (SSRI) are common psychotropic medications prescribed both in the community and hospital settings for patients with mental health conditions. We report the case of paroxetine-induced black hairy tongue (BHT) and necrotizing sialometaplasia (NS), presenting in a 27-year-old patient on long term SSRIs for her long-standing mental health. Both of these lesions were managed conservatively.

## Case presentation

**Fig. 1:**
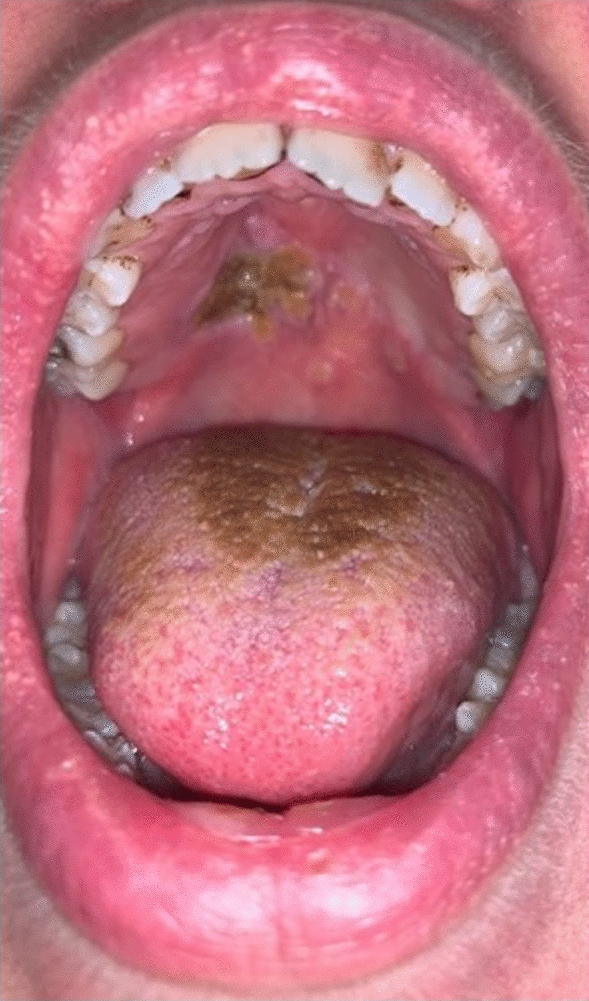
Inflamed and ulcerated palate with debris deposits on a brown hairy tongue

**Fig. 2:**
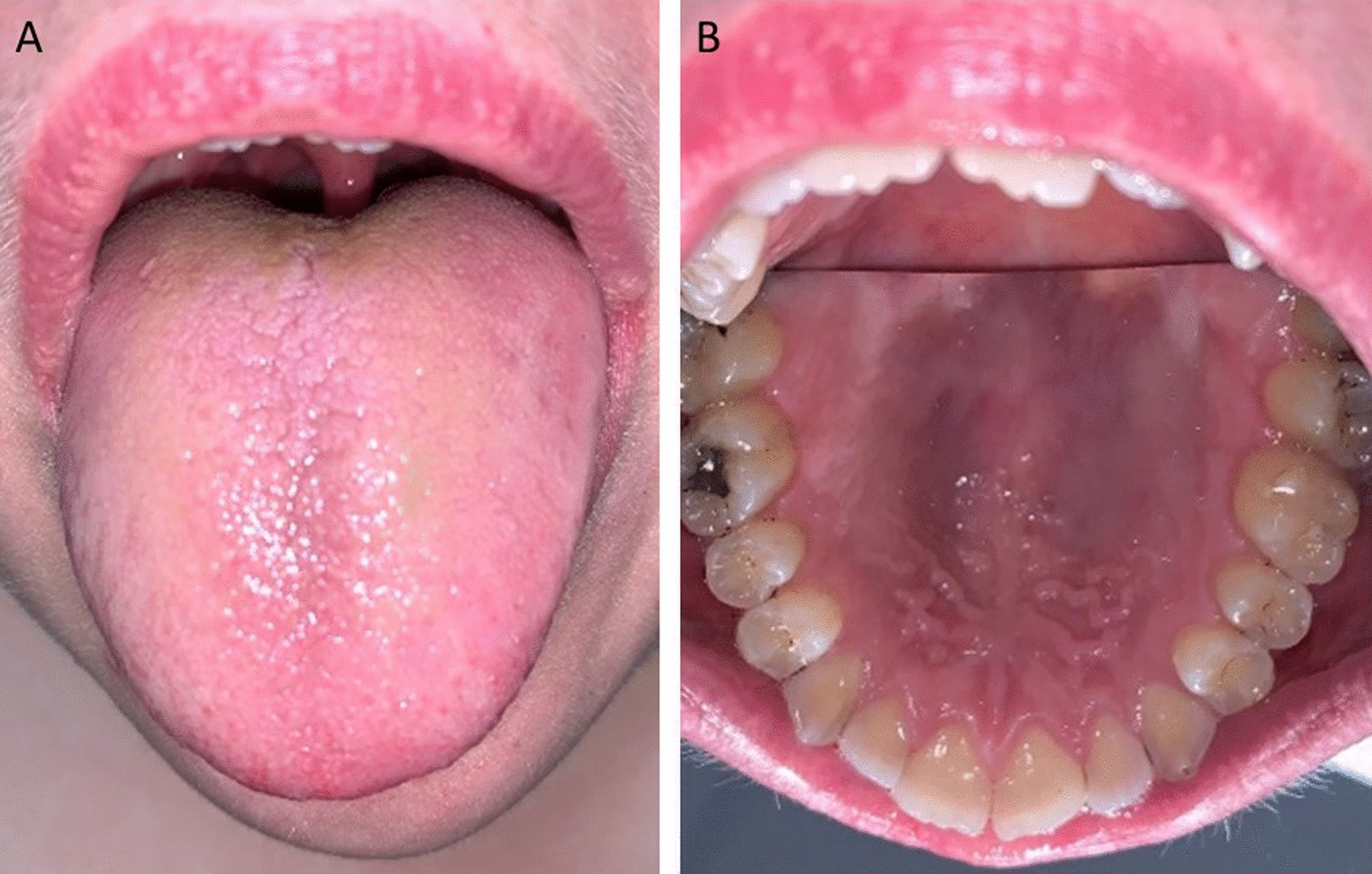
Images of the patient’s oral cavity following the withdrawal of the selective serotonin reuptake inhibitor; **A** dorsum of the tongue, **B** palate

A 27-year-old Caucasian (British) lady presented with a 4-month history of recurrent ulcerations affecting her soft palate. She was a light smoker, a teetotaler, and she had a background of von Willebrand’s disease, depression, treatment-resistant generalized anxiety disorder, social phobia, and hyperhidrosis. Her mental state was not improving despite various medications, including duloxetine, sertraline, venlafaxine, trazodone, and diazepam. She was started on paroxetine approximately 5 months prior to the development of the ulcers. Histopathology revealed nonspecific inflammation with no evidence of dysplasia. The lesions were managed conservatively; she was prescribed steroid mouthwashes and was advised to maintain good oral hygiene with regular salt water gargles and chlorhexidine mouthwashes. She returned after 19 months, following a quiescent period with a very inflamed and ulcerated palate, with heavy deposits of debris on a brown hairy tongue (Fig. 1). Her anxiety symptoms did not improve with paroxetine; hence, it was stopped. She was recommenced on venlafaxine and was introduced to buspirone. Interestingly, both of her oral lesions markedly improved following the withdrawal of paroxetine (Fig. 2).

## Discussion

BHT, also referred to as lingua villosa nigra, is a reversible benign disease characterized by elongated and hypertrophied filiform lingual papillae (longer than 3 mm), which are stained on the dorsum of the tongue. This condition has a prevalence of 0.6–11.3%. Despite being referred to as a black hairy tongue, yellow, green, and brown discolorations have previously been reported. The prevalence of BHT is higher in male patients, with a male:female ratio of 3:1. Other factors that predispose an individual to BHT include older age, poor oral hygiene, xerostomia, smoking, excessive coffee or black tea, alcohol, cancer, and certain drugs. Medications that have been associated with BHT include antimicrobials (penicillin, erythromycin, and doxycycline), anticholinergic drugs, psychotropic drugs (SSRIs, thiothixene hydrochloride, and benztropine mesylate), and antipsychotics (olanzapine) [1, 2].

The pathophysiology driving BHT remains unclear; literature suggests that BHT arises as a result of defective desquamation of the dorsal surface of the tongue, preventing normal debridement. This causes an accumulation of keratinized layers, leading to the elongation and hypertrophy of the filiform papillae. In addition, trapping of desquamating keratin and porphyrin-producing chromogenic microorganisms may contribute to the discoloration [2]. Furthermore, SSRIs have also been associated with hyperpigmentation. Serotonin has been shown to induce melanin production in melanocytes [3].

Patients with BHT are normally asymptomatic, although they may present with halitosis, dysgeusia, nausea, gagging, xerostomia, and burning sensation. Diagnosis can be made by clinical examination. These lesions can be debrided or managed conservatively (removal of causative agent and good oral hygiene) [2].

NS, on the other hand, is a benign, rare, and self-limiting reactive inflammatory disorder involving a minor salivary gland. NS is normally unilateral, with the hard palate being the most common site of occurrence. Lesions have also been reported on the lips, tongue, floor of the mouth, retromolar trigone, hypopharynx, and tonsillar pillars. Predisposing factors proposed in literature include smoking, alcohol, local trauma, local vasoconstriction and ischemia, drugs (excessive topical nonsteroidal antiinflammatory drugs), vasculitis (granulomatosis with polyangiitis), and purging in bulimia nervosa [4].

**Fig. 3:**
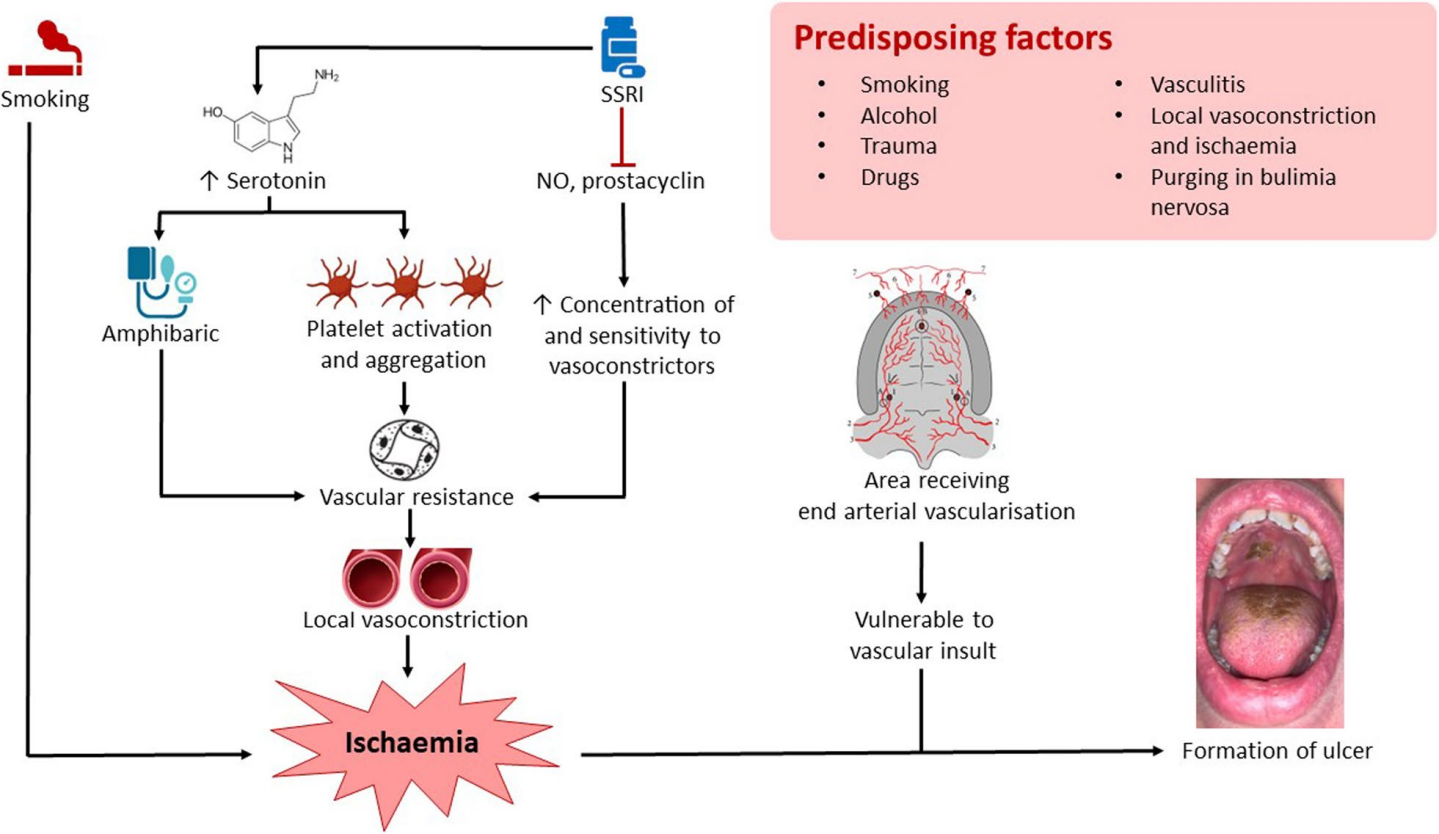
Pathophysiology driving necrotizing sialometaplasia, in particular, risk factors present in our patient

In the case of our patient, although she was a light smoker, local vasoconstriction seemed to be the likely cause of NS. The use of SSRIs increases plasma serotonin levels. Serotonin is an amphibaric agent that has both vasoconstrictor and vasodilator properties. Serotonin also contributes to platelet activation and aggregation [5]. SSRIs have been shown to inhibit nitric oxide (NO), a vasodilator, and prostacyclin, consequently increasing the concentrations of and sensitivity to local vasoconstrictors, increasing vascular resistance and causing ischemia [6]. In addition, experiments done on damaged blood vessels *in vivo* showed that serotonin amplified platelet aggregation response and induced localized vasoconstriction to facilitate vessel repair [5]. An experiment, which investigated the actions of different antidepressants on noradrenaline-induced vasoconstriction in rat isolated aorta, showed that compared with tricyclic antidepressants and trazodone, SSRIs had potent inhibitory effects on depolarization-induced vasoconstriction by blocking Ca^2+^ entry through L-type Ca^2+^ channels [7]. This may imply that our patient’s concurrent use of trazodone with an SSRI may have been contributing to the development of NS. In our patient, NS occurred along the midline of her soft palate, an area which receives end arterial vascularization from the vascular anastomoses between the descending palatine artery and ascending palatine artery, as well as the vascular anastomoses between the greater palatine artery and anterior palatine artery [8]. These end arteries are more vulnerable to vascular insult and ischemia (Fig. 3).

Patients with NS commonly describe having a lesion that begins as a localized erythematous swelling or nodule that subsequently breaks down and transforms into an ulcer with a necrotic base. They may be asymptomatic or may describe experiencing discomfort or pain over the site of the lesion. Diagnosis can often be made clinically; however, the appearance of NS may mimic oral malignancies (mucoepidermoid carcinoma, adenoid cystic carcinoma, and squamous cell carcinoma), mucormycosis, soft palate subacute necrotizing sialadenitis, and Whartin’s tumor. Diagnosis may also be facilitated by radiological imaging with cone beam computer tomography as well as immunohistochemical staining to identify residual myoepithelial cells by looking for calponin, smooth muscle actin, and cytokeratin-7 expression in NS. Importantly, NS has the potential to induce squamous metaplastic transformation of acinar cells; hence, timely and accurate diagnosis is important. NS is usually self-limiting and can be managed conservatively. Alternative treatment options include low-level laser therapy or photobiomodulation [5].

## Conclusion

This case demonstrates the need to consider BHT and NS as differential diagnoses in patients with relevant risk factors presenting with oral lesions. Diagnosis of BHT and NS can be made clinically. Timely and accurate diagnosis is important owing to the fact that NS may closely resemble more sinister pathology. BHT and NS can both be managed conservatively by removal of the causative factor and maintaining good oral hygiene. Clinicians may also consider including BHT and NS as rare side effects of SSRIs when offering medication counseling.

## Supplementary Information

Additional file 1.

## Data Availability

Not applicable.

## Abbreviations

BHT: Black hairy tongue
NO: Nitric oxide
NS: Necrotizing sialometaplasia
SSRI: Selective serotonin reuptake inhibitor
